# Association of Registered Nurse Staffing With Mortality Risk of Medicare Beneficiaries Hospitalized With Sepsis

**DOI:** 10.1001/jamahealthforum.2022.1173

**Published:** 2022-05-27

**Authors:** Jeannie P. Cimiotti, Edmund R. Becker, Yin Li, Douglas M. Sloane, Scott K. Fridkin, Anna Beth West, Linda H. Aiken

**Affiliations:** 1 Nell Hodgson Woodruff School of Nursing, Emory University, Atlanta, Georgia; 2 Rollins School of Public Health, Emory University, Atlanta, Georgia; 3 School of Nursing, University of Pennsylvania, Philadelphia; 4 School of Medicine, Emory University, Atlanta, Georgia; 5 VA Quality Scholars Program, Atlanta VA Health Care System, Atlanta, Georgia

## Abstract

**Importance:**

Sepsis is a major physiologic response to infection that if not managed properly can lead to multiorgan failure and death. The US Centers for Medicare & Medicaid Services (CMS) requires that hospitals collect data on core sepsis measure Severe Sepsis and Septic Shock Management Bundle (SEP-1) in an effort to promote the early recognition and treatment of sepsis. Despite implementation of the SEP-1 measure, sepsis-related mortality continues to challenge acute care hospitals nationwide.

**Objective:**

To determine if registered nurse workload was associated with mortality in Medicare beneficiaries admitted to an acute care hospital with sepsis.

**Design, Setting, and Participants:**

This cross-sectional study used 2018 data from the American Hospital Association Annual Survey, CMS Hospital Compare, and Medicare claims on Medicare beneficiaries age 65 to 99 years with a primary diagnosis of sepsis that was present on admission to 1 of 1958 nonfederal, general acute care hospitals that had data on CMS SEP-1 scores and registered nurse workload (indicated by registered nurse hours per patient day [HPPD]). Patients with sepsis were identified based on 29 *International Classification of Diseases, 10th Revision, Clinical Modification* (*ICD-10-CM*) codes. Data were analyzed throughout 2021.

**Exposures:**

SEP-1 score and registered nurse staffing.

**Main Outcomes and Measures:**

The patient outcome of interest was mortality within 60 days of admission. Hospital characteristics included number of beds, ownership, teaching status, technology status, rurality, and region. Patient characteristics included age, sex, transfer status, intensive care unit admission, palliative care, do-not-resuscitate order, and a series of 29 comorbid diseases based on the Elixhauser Comorbidity Index.

**Results:**

In total, 702 140 Medicare beneficiaries (mean [SD] age, 78.2 [8.7] years; 360 804 women [51%]) had a diagnosis of sepsis. The mean SEP-1 score was 56.1, and registered nurse HPPD was 6.2. In a multivariable regression model, each additional registered nurse HPPD was associated with a 3% decrease in the odds of 60-day mortality (odds ratio, 0.97; 95% CI 0.96-0.99) controlling for SEP-1 score and hospital and patient characteristics.

**Conclusions and Relevance:**

The results of this cross-sectional study suggest that hospitals that provide more registered nurse hours of care could likely improve SEP-1 bundle compliance and decrease the likelihood of mortality in Medicare beneficiaries with sepsis.

## Key Points

**Question:**

Is registered nurse workload associated with mortality among Medicare beneficiaries who are admitted to an acute care hospital with a diagnosis of sepsis?

**Findings:**

In this cross-sectional study of 1958 acute care hospitals and 702 140 Medicare beneficiaries with a diagnosis of sepsis, an increase in registered nurse hours per patient day was associated with a 3% decrease in 60-day mortality in these older adults, a finding that was statistically significant.

**Meaning:**

The study results suggest that the hours of care provided by registered nurses is likely associated with the outcomes of patients with a diagnosis of sepsis.

## Teaser

This cross-sectional study examines the association of registered nurse workload with mortality in Medicare beneficiaries admitted to an acute care hospital with sepsis.

## Introduction

Sepsis develops when the body’s response to an infection becomes dysregulated and organ dysfunction ensues.^1^ Sepsis is a major health problem in the US, and there are more than 1.7 million cases of sepsis diagnosed annually.^2^ In addition, sepsis is a leading cause of death, and it has been identified as one of the most expensive conditions treated by hospitals nationwide.^2,3,4,5,6,7^ Among Medicare beneficiaries, sepsis-related mortality and the costs associated with the inpatient and outpatient treatment of sepsis continue to escalate.^4^

The treatment of sepsis has evolved substantially during the last 20 years. The first standard treatment for sepsis resulted in what has been termed *early goal-directed therapy*.^8^ With the increase in evidence-based treatment strategies for sepsis, the Surviving Sepsis Campaign was launched, which represented a collaborative effort between the European Society of Intensive Care Medicine, Society of Critical Care Medicine, International Sepsis Forum, and Institute for Healthcare Improvement. Since its inception, the Surviving Sepsis Campaign has published and updated clinical practice guidelines, including bundles of care that outline actions to be completed within a specific period after a patient receives a diagnosis of sepsis.^9,10^

The US Centers for Medicare & Medicaid Services (CMS) implemented the Sepsis Core Measure SEP-1: The Severe Sepsis and Septic Shock Management Bundle based on a National Quality Forum–endorsed measure.^11,12^ Hospitals are evaluated on an all-or-nothing basis for the SEP-1 measure; ie, all components of the measure must be met for a hospital to be compliant with the measure for a patient with sepsis.^11^ To meet those criteria, recognizing sepsis early is essential, and communication among clinicians cannot be overlooked. Although the benefit of nurse-driven sepsis treatment tools has been reported,^13,14,15,16,17^ it is nurse-physician communication that has been identified as a necessary component to support the improvement that is needed in sepsis care.^18^

To be compliant with the SEP-1 early management bundle, all bundle components must be implemented within 3 hours in the presence of a systemic inflammatory response (temperature of >101° F or <96.8° F, heart rate >90 beats/minute, respirations >20 breaths/minute, and white blood cell count of >12 000 or <4000/μL, or >10% bandemia) or a diagnosis of suspected severe sepsis or septic shock. These components include not only serum lactate levels and blood cultures, but also antibiotics and crystalloid fluids. Several studies have reported that the timely administration of antibiotics^19^ and intravenous fluids are associated with improved SEP-1 scores and patient outcomes, and these are treatments that are typically delivered by nurses. Nurse-initiated sepsis treatment and communication protocols in the emergency department and inpatient acute care units have been associated with improvements in SEP-1 bundle compliance and a significant decrease in sepsis-related mortality.^14,17,18^ In addition, the organizational commitment of leadership, implementation of early sepsis or systemic inflammatory response syndrome screening tools, sepsis screening and response protocols, and education and training of nurses have all been associated with a significant decrease in sepsis-related mortality in Medicare beneficaries.^20^

Some have identified the workload of nurses as the primary mechanism that prevents the rapid initiation of antibiotic treatment to patients with a diagnosis of sepsis^21^ and that an adequate number of nurses are necessary to improve SEP-1 bundle compliance.^18^ In recent work, we found that nurse staffing was associated with 60-day mortality in patients with sepsis in hospitals across New York State.^22^ In the current study, we examined a nationwide sample of hospitals and Medicare beneficiaries with a diagnosis of sepsis to estimate the association between the hours of care provided by registered nurses and 60-day mortality and project the number of lives that could be saved by increasing the number of hours of nursing care.

## Methods

### Study Design and Sample

This was a cross-sectional study of 3 linked data sources: (1) the 2018 American Hospital Association (AHA) Annual Survey on hospital size, teaching and technology status, and nurse staffing; (2) 2018 hospital performance on the SEP-1 bundle for timely and effective sepsis care from the CMS Hospital Compare; and (3) 2018 patient characteristics from the Medicare Provider Analysis and Review (MedPAR) file on all Medicare fee-for-service acute care hospitalizations.

Hospitals were included if their data on SEP-1 scores were available through the CMS Hospital Compare and data on nurse staffing were available through AHA. The sample of hospitals included 1958 general acute care hospitals nationwide. Federal hospitals and hospitals that did not report data on SEP-1 scores and nurse staffing were excluded.

Patients were included if they were Medicare beneficiaries age 65 to 99 years and admitted to an acute care hospital with a principal diagnosis of sepsis. Patients with severe sepsis and septic shock present on admission were identified based on a series of 29 *International Classification of Diseases, 10th Revision, Clinical Modification* (*ICD-10-CM*) principal diagnosis codes (eTable 1 in the Supplement). Additional patient characteristics included age, sex, transfer status, intensive care unit (ICU) admission, palliative care, do-not-resuscitate (DNR) order, sepsis-related Medicare Severity Diagnosis Related Groups (MSDRG), and 29 comorbid diseases based on the Elixhauser Comorbidity Index. The final sample included 702 140 Medicare beneficiaries with sepsis.

This study was reviewed and approved by the institutional review board at Emory University, which waived informed consent based on the use of deidentified data, and it followed the Strengthening the Reporting of Observational Studies in Epidemiology (STROBE) reporting guidelines for cross-sectional studies.

### Outcomes and Exposures

The primary outcome of interest was 60-day mortality in Medicare beneficiaries with a diagnosis of sepsis. The key exposure variables of interest were SEP-1 score and direct care registered nurse staffing. The SEP-1 score of hospital performance on timely and effective sepsis care was obtained from the CMS Hospital Compare. The SEP-1 score represents the percentage of patients who received appropriate care for severe sepsis or septic shock. The care bundle includes serum lactate levels, collection of blood cultures, and the delivery of broad-spectrum antimicrobial therapy within 3 hours of sepsis onset for individuals with severe sepsis. Treatment with intravenous fluids within 3 hours of onset and vasopressors within 5 hours and repeated volume assessments within 6 hours are additionally required for patients with a diagnosis of septic shock. The SEP-1 score has a possible range of 0% to 100%. The measure of direct care registered nurse staffing was based on previous work and computed as registered nurse hours per patient day (HPPD),^23^ in which the total number of full-time–equivalent registered nurses was multiplied by 1768 hours per year and divided by the total adjusted patient days.

### Covariates

Hospital characteristics were those available through AHA. The size of a hospital was defined as fewer than 100 beds, 101 to 250 beds, 251 to 500 beds, or more than 500 beds; hospital ownership was public, nonprofit, or for profit. Hospital academic status was defined as nonteaching, minor teaching (0-4 medical residents per bed), or major teaching (≥4 medical residents per bed); and technology status was determined by whether a hospital performed transplant surgery of the heart, kidney, lungs, and liver or adult cardiac surgery. Hospital location was classified as metropolitan, micropolitan, or rural, and the 9 geographic Census Regions of the US (New England, Mid Atlantic, South Atlantic, East North Central, East South Central, West North Central, West South Central, Mountain, and Pacific) were included. In addition, a variable was created to identify hospitals with fewer than 10 sepsis cases during the year of analysis.

Data on patient characteristics obtained from the Medicare Provider Analysis and Review included age, sex, transfer in from another hospital, an ICU admission, palliative care, DNR, and a set of binary measures that included the 29 comorbid diseases identified through the Elixhauser Comorbidity Index. The level of sepsis severity was defined based on 5 MSDRGs: 853, infectious disease with major complication or comorbidity; 854, infectious disease with a complication or comorbidity; 870, severe sepsis requiring mechanical ventilation for longer than 96 hours; 871, severe sepsis without receipt of mechanical ventilation for longer than 96 hours without major complication or comorbidity; 872, severe sepsis without receipt of mechanical ventilation longer than 96 hours with major complication or comorbidity; and other.

### Statistical Analysis

Descriptive statistics were used to examine all hospital and patient characteristics. A logistic regression model was fit to examine the association between registered nurse HPPD and 60-day mortality. The fully adjusted regression model included all hospital and patient characteristics shown in Table 1 and Table 2, and robust standard errors were used to account for the clustering of patients within hospitals.. Projected 60-day mortality was computed based on all hospitals meeting hypothetical minimum levels of registered nurse HPPD. All analyses were conducted using Stata, version 15.1 (StataCorp), and significance was set at *P *<* *.05.

**Table 1.** Characteristics of 1958 Study Hospitals That Submitted SEP-1 Scores

Characteristic	No. (%)
SEP-1, mean (SD), score^a^	56.1 (15.6)
Registered nurse, mean (SD), HPPD	6.2 (2.3)
Assistive personnel, mean (SD), HPPD	1.7 (1.0)
Intensivist on staff	1295 (66)
No. of beds	
≤100	627 (32)
101-250	657 (34)
251-500	455 (23)
>500	219 (11)
Ownership	
Public	272 (14)
Nonprofit	1454 (74)
For profit	232 (12)
Teaching status	
Nonteaching	1413 (72)
Minor	329 (17)
Major	216 (11)
High technology status	832 (43)
Rurality	
Metropolitan	1486 (76)
Micropolitan	335 (17)
Rural	137 (7)
Region	
New England	94 (5)
Mid Atlantic	260 (13)
South Atlantic	330 (17)
East North Central	299 (15)
East South Central	121 (6)
West North Central	216 (11)
West South Central	326 (17)
Mountain	96 (5)
Pacific	216 (11)

Abbreviations: HPPD, hours per patient day; SEP-1, Centers for Medicare & Medicaid Services core measure for sepsis, The Severe Sepsis and Septic Shock Management Bundle.

^a^ SEP-1 scores range from 0% to 100% and indicate the percentage of patients who received the Centers for Medicare & Medicaid Services recommended care for severe sepsis and septic shock.

**Table 2.** Characteristics of 702 140 Patients With a Principal Diagnosis of Sepsis

Characteristic	No. (%)
Age, mean (SD), y	78.2 (8.7)
Sex	
Female	360 804 (51)
Male	341 336 (49)
Transferred in	37 453 (5)
Intensive care unit admission	326 003 (46)
Palliative care	84 106 (12)
Do not resuscitate	188 957 (27)
Medicare severity-diagnosis related group	
853 Infectious disease with MCC	63 384 (9)
854 Infectious disease with CC	14 965 (2)
870 Severe sepsis with MV >96 h	19 578 (3)
871 Severe sepsis without MV >96 h, without MCC	469 683 (67)
872 Severe sepsis without MV >96 h, with MCC	128 933 (18)
Other DRGs^a^	5597 (1)
Elixhauser comorbidities^b^	
Hypertension, complicated	548 410 (78)
Fluid and electrolyte disorders	442 223 (63)
Chronic pulmonary disease	230 076 (33)
Congestive heart failure	230 133 (33)
Kidney failure	215 936 (31)
Deficiency anemias	202 092 (29)
Diabetes with complication	198 325 (28)
Hypothyroidism	137 775 (20)
Neurological disorders	123 295 (18)
Weight loss	113 765 (16)
Obesity	105 272 (15)
Depression	92 327 (13)
Coagulopathy	91 744 (13)
Diabetes	85 050 (12)
Valvular disease	74 427 (11)
Peripheral vascular disease	73 404 (11)
60-d Mortality	182 346 (26)

Abbreviations: CC, complication or comorbidity; DRG, diagnosis-related group; MCC, major complication or comorbidity; MV, mechanical ventilation.

^a^ Other DRGs include 969, 970, 974, 975, and 976.

^b^ Elixhauser comorbidities shown are those represented by 10% or more of patients.

## Results

The characteristics of the study hospitals are shown in Table 1. A total of 1284 hospitals (66%) were small or moderately small based on number of beds; large (219 [11%]) and moderately large (455 [23%]) hospitals were slightly less represented. Most hospitals were nonprofit (1454 [74%]) and nonteaching (1413 [72%]), and 832 hospitals (43%) were high-technology facilities. Most hospitals were located in metropolitan areas (1486 [76%]), and although they were somewhat equally distributed geographically, New England, East South Central, and Mountain regions were less represented at 5%, 6%, and 5%, respectively. The mean (SD) hospital SEP-1 score was 56.1 (15.6).

Hospitals provided a mean (SD) of 6.2 (2.3) registered nurse HPPD. The large hospitals, or those with more than 500 beds, provided more registered nurse HPPD (7.7), as did for-profit hospitals (6.4) and major teaching hospitals (7.4). Hospitals that were high technology (HPPD, 7.0), located in metropolitan areas (HPPD, 6.5), and located in the Pacific region of the US (HPPD, 8.1) all provided more registered nurse HPPD compared with the overall average hours.

The characteristics of the 702 140 patients included in this study are described in Table 2. The mean (SD) age of patients was 78 (8.7) years, 360 804 (51%) were female, 37 453 (5%) transferred in from another acute care hospital, and 326 003 (46%) were admitted to an ICU. A total of 84 106 patients (12%) received palliative care, and 188 957 (27%) had a DNR order. Based on MSDRG, most patients (469 683 [67%]) had severe sepsis without a major complication or comorbidity (MSDRG 871), followed by 128 933 patients (18%) with severe sepsis with a major complication or comorbidity (MSDRG 872). The most common comorbidities included complicated hypertension (548 410 [78%]), fluid and electrolyte disorders (442 223 [63%]), chronic pulmonary disease (230 076 [33%]), congestive heart failure (230 133 [33%]), and kidney failure (215 936 [31%]). Overall, 182 346 patients (26%) died within 60 days of admission.

Table 3 shows the odds ratios (ORs) from the multivariable regression model, in which exposure to a 10% increase in SEP-1 score was associated with a 2% decrease in the odds of 60-day mortality (OR, 0.98; 95% CI, 0.97-0.99; *P* = .01) while controlling for hospital and patient characteristics. Exposure to each additional registered nurse HPPD was associated with a 3% decrease in the odds of 60-day mortality (OR, 0.97; 95% CI, 0.96-0.99; *P* < .001), and an intensivist on staff was associated with a 16% decrease in the odds of 60-day mortality (OR, 0.84; 95% CI, 0.79-0.89; *P* < .001) while controlling for SEP-1 score and hospital and patient characteristics (eTable 2 in the Supplement).

**Table 3.** Multivariable Association of SEP-1 Score and Nurse Staffing With Patient Mortality

Variable	Unadjusted^a^		Adjusted^b^	
	Odds ratio (95% CI)	*P* value	Odds ratio (95% CI)	*P* value
SEP-1 score	0.97 (0.96-0.98)	<.001	0.98 (0.97-0.99)	.01
Registered nurse HPPD	0.99 (0.99-1.00)	.09	0.97 (0.96-0.99)	<.001
Assistive personnel HPPD	NA	NA	1.00 (0.98-1.03)	.86
Intensivist on staff			0.84 (0.79-0.89)	<.001
No. of beds				
≤100			1 [Reference]	NA
101-250			1.11 (1.05-1.18)	<.001
251-500			1.15 (1.07-1.24)	<.001
>500			1.05 (0.96-1.15)	.29
High technology status			0.96 (0.91-1.01)	.09
Teaching status				
Nonteaching			1 [Reference]	NA
Minor			0.99 (0.94-1.04)	.70
Major			0.95 (0.89-1.01)	.10
Ownership				
Public			1 [Reference]	NA
Nonprofit			0.88 (0.82-0.94)	<.001
For profit			1.06 (0.97-1.15)	.18

Abbreviations: CMS, Centers for Medicare & Medicaid Services; HPPD, hours per patient day; NA, not applicable; SEP-1, CMS core sepsis measure, The Severe Sepsis and Septic Shock Management Bundle.

^a^ The unadjusted odds ratios are from separate bivariate regression models that estimated the association of SEP-1 score and registered nurse HPPD with mortality with no controls.

^b^ The adjusted regression model included all hospital and patient covariates shown in Table 1 and Table 2, and robust standard errors were used to account for the clustering of patients within hospitals. Hospital characteristics were included rurality and region. Patient characteristics included age, sex, transfer status, palliative care, do-not-resuscitate order, 29 Elixhauser comorbidities, and a dummy variable for each sepsis diagnosis-related group.

The projected deaths avoided if all hospitals staffed at different levels of registered nurse HPPD and the change in the number of sepsis-related deaths within 60 days of admission are shown in Table 4. These findings suggest that if all hospitals were staffed at 6 registered nurse HPPD or higher there could be 1266 fewer deaths. This projected trend continued, in which if all hospitals were staffed at 9 registered nurse HPPD or higher, there could be 6360 avoided patient deaths.

**Table 4.** Projected Deaths Avoided Based on a Minimum of Registered Nurse Hours

Nurse hours	Deaths	Change
Observed RN HPPD	182 346	NA
6 RN HPPD	181 080	−1266
7 RN HPPD	179 797	−2549
8 RN HPPD	178 040	−4306
9 RN HPPD	175 986	−6360

Abbreviations: HPPD, hours per patient day; NA, not applicable; RN, registered nurse.

## Discussion

In this cross-sectional study containing a large sample of hospitals, we found that overall SEP-1 compliance scores were low nationwide and that many patients died of sepsis. Despite the fact that sepsis definitions and the recommended treatments have changed during the past 2 decades, little success has been noted in the treatment of sepsis.^24,25^ Based on our analysis of hospitals and patients nationwide, the study findings suggest that nurse workload is an overlooked and underused aspect of the treatment bundle for patients with a diagnosis of sepsis.

Research on sepsis is extensive, and many have reported that compliance with the SEP-1 bundle has not been associated with its desired outcome.^24,25,26,27,28^ In hospitals nationwide, SEP-1 performance has been associated with hospital structural characteristics,^29,30^ with variability in rates of bundle compliance that range from 9% to 69%.^31^ Delays in testing serum lactate levels and the delivery of antibiotics are widespread across settings, with delays of 68% and 45% reported for medical units and ICUs, which result in roughly a 2-hour delay in antibiotic administration.^32^ It has been hypothesized that system factors likely contribute to the reported delays in the delivery of treatment with antibiotics to patients with severe sepsis,^29,33^ and that improvements to system factors are necessary to facilitate physician prescribing practices, the verification process required of pharmacists, and the administration of antibiotics by nurses to patients with sepsis.^33^

Recognizing sepsis early is essential, and the effect of interprofessional teamwork cannot be overlooked. It has been reported that nurse-physician communication and collaboration are necessary components to improve sepsis care.^18^ Research suggests that nurse-physician communication was associated with a 4% decrease in the likelihood that patients would develop catheter-associated sepsis.^34^ Differences in the association between nurse and physician staffing have also been reported, in which each additional step-down patient per nurse was associated with a 0.53 increase in sepsis.^35^ An increase in hospitalist and physician HPPD was associated with an increase in sepsis (0.42 and 0.22, respectively); however, an increase in intensivist HPPD was associated with a decrease (−0.37) in sepsis. The present study produced similar findings, in which the presence of an intensivist was associated with a substantial decrease in the odds of 60-day mortality in patients with sepsis. Additionally, the presence of an intensivist strengthened the association between registered nurse HPPD and reduced sepsis-related mortality, which could be a result of interprofessional communication. These findings highlight the important relationships that exist between nurses and physicians and the likelihood that nurses might report findings to 1 group of physicians (eg, intensivists) over another (eg, hospitalist or physician), and it supports the importance of communication between all clinicians.

The initiation of nurse-led sepsis protocols has been associated with obtaining timely serum lactate levels and blood cultures; however, the timely delivery of antibiotics and fluid administration continue to be suboptimal.^36^ In a nationwide survey of emergency departments, 58% of physician directors and 48% of nurse managers identified nurse staffing as the primary cause of delays in treating sepsis.^37^ In a recent article, we reported that each additional patient added to a nurse’s workload was associated with a 12% increase in the likelihood of in-hospital death, a 7% increase in 60-day mortality and 60-day readmission, and longer lengths of stay in patients with sepsis.^22^ Despite previous research that examined large samples of medical and surgical patients using different measures of nurse staffing (registered nurse HPPD and patients per nurse) and provided landmark evidence that nurse staffing was associated with an increase in mortality and failure-to-rescue,^38,39^ to our knowledge, the present study is the first to examine nurse workload and sepsis outcomes in a nationwide sample of Medicare beneficiaries.

Nurse workload has been identified as a factor that contributes to delays or omission in medication administration on medical-surgical and intensive care units.^40^ There has also been ongoing evidence to suggest an association between nurse workload and the spread of infectious diseases in acute care hospitals.^41,42,43,44,45,46,47^ Some have reported that despite the implementation of care bundles, infections continue to affect health care systems, most likely because of nurse workload, which has been identified as a barrier to bundle adherence.^48,49^ Clinicians have reported that there are several challenges that hinder compliance with care bundles.^49^ These include the fact that staff are too busy, reported staff shortages, the lack of staff engagement, and the added workload associated with the implementation of care bundles. Much of the research provided by this group of authors and the work of others provides evidence that there is a need for registered nurse staffing to be factored into all care bundles across practice settings.

### Limitations

This study had a few limitations. First, this study was cross-sectional, and by design it was not possible to determine a causal association between registered nurse HPPD and sepsis-related 60-day mortality. However, there are multiple studies with prospectively collected data that have reported an association between nurse staffing and patient outcomes, such as mortality and nonmortality adverse events.^50,51^ Second, because of our interest in nurse workload, we only included hospitals that reported data on facility-wide nurse staffing to AHA. The AHA Annual Survey is a commonly used data source on nurse workload, and several landmark studies have used AHA nurse staffing data to examine patient outcomes.^39,52,53^ Furthermore, in this study, many hospitals included in the analyses were large and represented many nurses and patients; therefore, we do not consider this to be a major limitation. Third, the patient population was restricted to Medicare beneficiaries; however, there is evidence to show that older adults are the patients most likely to develop sepsis and subsequent sepsis-related outcomes.^54,55,56^ Finally, the source for SEP-1 bundle compliance was those scores that were publicly available through the CMS Hospital Compare. Because SEP-1 is an all-or-nothing measure, we know that certain hospitals were penalized for what could have been a slight delay in the implementation of just 1 component, whereas other hospitals could have been less compliant in multiple aspects of the SEP-1 bundle. Regardless of the violation in SEP-1 bundle compliance, it is likely that an undetermined number of hospital cases were not included in the CMS Hospital Compare; however, these are the only data available on SEP-1 compliance. Despite these limitations, to our knowledge, we provided the first nationwide report on SEP-1 compliance, registered nurse staffing, and 60-day mortality in Medicare beneficiaries.

## Conclusions

The results of this cross-sectional study suggest that hospitals nationwide continue to struggle with SEP-1 bundle compliance. We found that nurse workload was associated with 60-day mortality in Medicare beneficiaries who were admitted to an acute care hospital with sepsis. As we redefine the sepsis bundle, it is imperative that we include the workload of nurses and other clinicians and promote a care environment that fosters interprofessional communication. Not doing so will place patients at increased risk of sepsis-related mortality and nonmortality adverse events.

## Notes

Supplement. **eTable 1. **International Classification of Diseases, 10th Revision (ICD-10) Principal Diagnosis Codes for Sepsis **eTable 2. **Adjusted Effects of Registered Nurse Staffing and SEP-1 Score on Patient Mortality
