# Supplementary material for: Association of Registered Nurse Staffing With Mortality Risk of Medicare Beneficiaries Hospitalized With Sepsis
Source: JAMA Health Forum. 2022 May 27;3(5):e221173. doi: 10.1001/jamahealthforum.2022.1173 (PMC9142874; doi:10.1001/jamahealthforum.2022.1173)
Supplement: Supplement. — eTable 1. International Classification of Diseases, 10th Revision (ICD-10) Principal Diagnosis Codes for Sepsis eTable 2. Adjusted Effects of Registered Nurse Staffing and SEP-1 Score on Patient Mortality [file jamahealthforum-e221173-s001.pdf]

Supplemental Online Content

Cimiotti JP, Becker ER, Li Y, et al. Association of registered nurse staffing with mortality risk of Medicare beneficiaries hospitalized with sepsis. *JAMA Health Forum*. 2022;3(5):e2211733. doi:10.1001/jamahealthforum.2022.1173

eTable 1. International Classification of Diseases, 10th Revision (ICD-10) Principal Diagnosis Codes for Sepsis

eTable 2. Adjusted Effects of Registered Nurse Staffing and SEP-1 Score on Patient Mortality

This supplemental material has been provided by the authors to give readers additional information about their work.

© 2022 Cimiotti JP et al. *JAMA Health Forum.*

eTable 1. International Classification of Diseases, 10th Revision (ICD-10) Principal Diagnosis Codes for Sepsis

ICD-10	Description
A021	*Salmonella* sepsis
A227	*Anthrax* sepsis
A267	*Erysipelothrix* sepsis
A327	Listerial sepsis
A400	Sepsis due to *Streptococcus*, group A
A401	Sepsis due to *Streptococcus*, group B
A403	Sepsis due to *Streptococcus pneumoniae*
A408	Other streptococcal sepsis
A409	Streptococcal sepsis, unspecified
A4101	Sepsis due to Methicillin susceptible *Staphylococcus aureus*
A4102	Sepsis due to Methicillin resistant *Staphylococcus aureus*
A411	Sepsis due to other specified *Staphylococcus*
A412	Sepsis due to unspecified *Staphylococcus*
A413	Sepsis due to *Hemophilus influenzae*
A414	Sepsis due to anaerobes
A4150	Gram-negative sepsis, unspecified
A4151	Sepsis due to *Escherichia coli* [E. coli]
A4152	Sepsis due to *Pseudomonas*
A4153	Sepsis due to *Serratia*
A4159	Other gram-negative sepsis
A4181	Sepsis due to *Enterococcus*
A4189	Other specified sepsis
A419	Sepsis, unspecified organism
A427	Actinomycotic sepsis
A5486	Gonococcal sepsis
B377	Candidal sepsis
R6520	Severe sepsis without septic shock
R6521	Severe sepsis with septic shock
R7881	Bacteremia

© 2022 Cimiotti JP et al. *JAMA Health Forum.*

eTable 2. Adjusted Effects of Registered Nurse Staffing and SEP-1 Score on Patient Mortality				
**Variable**	**Odds Ratio**	**SE**	**95% CI**	***p***
Sep-1 Score	0.98	0.01	0.97-0.99	<0.001
RN HPPD	0.97	0.01	0.96-0.99	<0.001
Assisted Personnel HPPD	1.00	0.01	0.98-1.03	0.86
Intensivist on staff	0.84	0.02	0.79-0.89	<0.001
Bed Size				
<=100	Reference			
101-250	1.11	0.03	1.05-1.18	<0.001
251-500	1.15	0.04	1.07-1.24	<0.001
>500	1.05	0.05	0.96-1.15	0.29
High technology status	0.96	0.02	0.91-1.01	0.09
Teaching status				
Non-teaching	Reference			
Minor	0.99	0.03	0.94-1.04	0.70
Major	0.95	0.03	0.89-1.01	0.10
Ownership				
Public	Reference			
Non-profit	0.88	0.03	0.82-0.94	<0.001
For-profit	1.06	0.04	0.97-1.15	0.18
Rurality				
Rural	Reference			
Micro	0.92	0.06	0.81-1.05	0.23
Metro	0.85	0.06	0.75-0.96	0.01
Region				
New England	Reference			
Mid Atlantic	1.40	0.06	1.29-1.53	<0.001

© 2022 Cimiotti JP et al. *JAMA Health Forum.*

South Atlantic	1.32	0.06	1.21-1.43	<0.001
East North Central	1.25	0.05	1.15-1.36	<0.001
East South Central	1.45	0.08	1.30-1.60	<0.001
West North Central	0.98	0.05	0.89-1.09	0.74
West South Central	1.35	0.06	1.24-1.47	<0.001
Mountain	0.97	0.06	0.86-1.09	0.59
Pacific	1.10	0.05	1.00-1.21	0.05
Age	1.02	0.00	1.02-1.02	<0.001
Gender, male	1.10	0.01	1.08-1.11	<0.001
Transferred in	1.22	0.03	1.16-1.28	<0.001
Intensive care unit admission	1.67	0.02	1.62-1.72	<0.001
Palliative care	10.33	0.29	9.78-10.9	<0.001
Do Not Resuscitate	3.30	0.05	3.21-3.40	<0.001
Medicare Severity-Diagnosis Related Group				
853 Infectious Disease with MCC	1.01	0.04	0.94-1.10	0.75
854 Infectious Disease with CC	0.23	0.01	0.20-0.26	<0.001
870 Severe Sepsis with MV>96h	2.73	0.11	2.52-2.96	<0.001
871 Severe Sepsis without MV>96h, without MCC	0.95	0.04	0.88-1.02	0.17
872 Severe Sepsis without MV>96h, with MCC	0.33	0.01	0.31-0.36	<0.001
Other DRG	Reference			
Elixhauser Comorbidities				
Congestive heart failure	1.44	0.01	1.42-1.46	<0.001
Valvular disease	1.02	0.01	1.00-1.05	0.04
Pulmonary circulation disease	1.45	0.04	1.38-1.52	<0.001
Peripheral vascular disease	1.30	0.02	1.38-1.52	<0.001
Paralysis	1.15	0.02	1.12-1.19	<0.001
Other neurological disorders	1.12	0.01	1.10-1.14	<0.001

© 2022 Cimiotti JP et al. *JAMA Health Forum.*

Chronic pulmonary disease	0.97	0.01	0.95-0.98	<0.001
Diabetes w/o chronic complications	1.03	0.01	1.02-1.06	0.01
Diabetes w/ chronic complications	1.08	0.01	1.06-1.10	<0.001
Hypothyroidism	0.93	0.01	0.92-0.95	<0.001
Renal failure	1.33	0.01	1.31-1.36	<0.001
Liver disease	1.57	0.02	1.52-1.62	<0.001
Peptic ulcer Disease x bleeding	0.97	0.03	0.92-1.02	0.24
Acquired immune deficiency syndrome	0.85	0.33	0.39-1.83	0.67
Lymphoma	1.42	0.03	1.35-1.48	<0.001
Metastatic cancer	4.19	0.07	4.06-4.32	<0.001
Solid tumor w/out metastasis	1.71	0.03	1.67-1.75	<0.001
Rheumatoid arthritis/collagen vas	0.98	0.02	0.95-1.01	<0.001
Coagulopathy	1.40	0.02	1.37-1.43	<0.001
Obesity	0.75	0.01	0.74-0.77	<0.001
Weight loss	1.71	0.02	1.67-1.75	<0.001
Fluid and electrolyte disorders	1.51	0.01	1.48-1.54	<0.001
Chronic blood loss anemia	1.09	0.04	1.02-1.18	<0.001
Deficiency Anemias	1.00	0.01	0.98-1.02	<0.001
Alcohol abuse	0.95	0.02	0.91-1.00	<0.001
Drug abuse	0.75	0.03	0.70-0.81	<0.001
Psychoses	0.93	0.02	0.89-0.97	<0.001
Depression	0.87	0.01	0.85-0.89	<0.001
Hypertension	0.81	0.01	0.80-0.83	<0.001

© 2022 Cimiotti JP et al. *JAMA Health Forum.*
